# Reservoir Compatibility and Enhanced Oil Recovery of Polymer and Polymer/Surfactant System: Effects of Molecular Weight and Hydrophobic Association

**DOI:** 10.3390/polym17101390

**Published:** 2025-05-18

**Authors:** Tao Liu, Xin Chen, Xiang Tang

**Affiliations:** 1 College of Earth Sciences and Engineering, Xi’an Shiyou University, Xi’an 710065, China; 2 Shaanxi Key Laboratory of Petroleum Accumulation Geology, Xi’an Shiyou University, Xi’an 710065, China; 3 College of Petroleum Engineering, Xi’an Shiyou University, Xi’an 710065, China; 4 China National Oil and Gas Exploration and Development Company Ltd., Beijing 100034, China

**Keywords:** molecular weight, hydrophobic association, matching, injectivity, mobility control, EOR

## Abstract

In this paper, four kinds of flooding systems, high-molecular-weight polymer (HMP), low-molecular-weight polymer (LMP), hydrophobic association polymer (HAP), and LMP/petroleum sulfonate (PS), are preferred. By comparing the static performance, their good basic characteristics as an oil displacement system are clarified. The application concentration range of the polymer solution is optimized and designed in combination with core injectivity experiments and mobility control theory. The oil displacement system and its injection volume have been optimized via three parallel core flooding experiments. The results show that the increase of the polymer molecular weight and the association will enhance the viscosity-increasing performance, viscosity stability, viscoelasticity, and hydrodynamic characteristic size of the solution. According to whether the injection pressure curve reaches equilibrium and the time required for equilibrium, the matching relationship between the polymer and the reservoir can be divided into plugging, flow difficulty and flow smoothly. Based on the mobility control theory, the minimum mobility of the target core occurs when the water saturation is 30–40%. Therefore, the polymer formulation for the application of combined cores with viscosities of 50 mD, 210 mD, and 350 mD is set at 1500 mg/L for LMP and 800 mg/L for MAP. HAP has the best profile improvement effect, but its lowest EOR is 9.68%, which mainly acts on high-permeability layers; LMP can produce more remaining oil in middle-permeability layers, and its EOR can reach 12.01%; LMP/PS can give full play to the oil displacement performance of the polymer and the oil washing ability of the surfactant, and its highest EOR is 21.32%. Meanwhile, the emulsification effect also makes the profile improvement last longer. According to the EOR efficiency and final oil recovery, the optimal injection volume of LMP/PS can be designed to be 0.6–0.7 PV.

## 1. Introduction

Based on the current trend and background of scientific and technological development, oil and gas resources will still be the mainstream energy in the coming decades [1,2]. With the progress of the exploration and development of oil and gas fields, unconventional oil reservoirs have become the main potential resources and research hotspots for the future energy supply [3,4,5,6]. However, conventional old oilfields have undergone secondary and tertiary oil recovery, and there is still a large amount of remaining oil buried underground [7,8]. Determining how to further improve the recovery factor or optimize the design of the tertiary oil recovery plan to increase the recovery factor can also obtain huge economic benefits. At present, chemical flooding is a tertiary oil recovery technology with a relatively mature theory and technology, and it plays a vital role in the sustained and stable growth of crude oil production in China [9,10,11].

Chemical flooding usually refers to adding chemical agents such as polymers and surfactants to the injected water to improve the performance of the oil displacement system to achieve the purpose of enhancing oil recovery [12,13,14,15]. Chemical flooding usually includes polymer flooding, polymer/surfactant (S/P) binary compound flooding, polymer/alkali/surfactant (A/S/P) triple-compound flooding, chemical profile control and water shutoff, etc. [16,17,18]. After more than 60 years of laboratory research and field experiments, polymer flooding has been successfully applied in China’s Daqing Oilfield, Xinjiang Oilfield, and Dagang Oilfield [19]. Polymer flooding mainly improves the water–oil mobility ratio by increasing the viscosity of the water phase to expand the swept volume. According to lab research, polymer flooding can increase recovery by 10–12% on the basis of water flooding [16,20]. Surfactants can reduce the oil–water interfacial tension and improve the oil-washing efficiency of sweep flooding. Surfactant flooding alone is seldom used in oilfields, and it is applied by compounding with polymers to form an S/P system. The S/P system can have both the mechanisms of expanding the swept volume and improving the oil-washing efficiency, and the enhanced recovery can exceed 20% [21,22]. Although ASP flooding can further improve the effect of the EOR, the addition of an alkali will increase the difficulty of producing water treatment and pollute the environment [23]. Therefore, under the theme of green and low carbon in today’s world, weak alkali ASP flooding and binary compound flooding are the main body of mine application [23,24]. The mixture system of the polymer and surfactant is prone to chromatographic separation in the reservoir, which affects the synergistic effect of the two [25,26]. Hydrophobic associative polymers introduce hydrophobic side chains into linear polymer molecules, which can greatly increase the solution viscosity through hydrophobic association and have interfacial activity [27,28,29]. Hydrophobic association polymer is considered a new type of chemical flooding system, and a large number of research results show that it has a good effect on enhancing oil recovery [20,29].

The key to the successful application of chemical flooding is that the chemical system can be injected into the reservoir and fully contact with crude oil. This refers to the eternal compatibility problem in the chemical flooding application process: to ensure the mobility improvement effect and good injectivity performance of the polymer solution at the same time. The viscosity of the oil displacement system is the most commonly used standard parameter for solution performance evaluation. However, for polymer solutions with the same viscosity, if their molecular weights are different, the performance of the solution will be quite different [30]. This is mainly caused by the difference in polymer molecular coils, which also directly affects the matching between the solution and the reservoir [31,32]. Based on the pore–throat size and the hydrodynamic characteristic size of the polymer solution, the matching relationship between the polymer and the reservoir can be primarily evaluated. Similarly, hydrophobically associating polymers have larger hydrodynamic feature sizes due to their more developed spatial structures, resulting in poor injectability [33]. Based on the premise of ensuring the polymer injectivity, increasing the molecular weight, concentration, or association strength of the polymer solution can effectively increase the oil recovery. Based on this idea, Zhu [34] proposed a stepwise viscosity reduction polymer injection method and showed its successful application in the Daqing Oilfield. Although the reduction of the polymer solution viscosity and molecular sizes can enhance its injectivity, the mobility control ability is reduced. Cai [35] and Zarin [36] proposed mobility design methods for polymer flooding and composite flooding, respectively, and calculated the minimum resistance factor required for the polymer to play a role in mobility control through phase–permeability curves. The basic role of the S/P composite flooding system is still due to the polymer, and the surfactant can fully contact with the crude oil after expanding the swept volume, and it can play on the advantage of washing oil. Meanwhile, the surfactant also has the functions of emulsification and solubilization, adding the Jiamin effect to further expand the swept volume and improving the wettability [37,38,39].

Although a lot of indoor research has been performed on polymer flooding and S/P flooding, there are still three deficiencies: (1) lack of systematic research on the influence of the molecular weight and association on the solution performance; (2) lack of a complete formula design method for chemical flooding system solutions, that is, the design of the upper and lower concentrations of polymer solutions; and (3) there is a lack of research on the optimization of the slug size of the oil displacement system.

Based on the above problems, low-molecular-weight polymers, high-molecular-weight polymers, hydrophobic association polymers (viscosity similar to high-molecular-weight polymers) and low-molecular-weight polymer/petroleum sulfonate systems are preferred due to the solution performance, oil displacement, and injection volume optimization. By comparing the viscosity-increasing properties, rheological properties, and viscosity stability of polymer solutions, the effects of the molecular weight and association on the basic properties of the solutions were compared. The matching relationship between the polymer solution and the core was evaluated by calculating the resistance factors through core injectivity experiments and explained through the hydrodynamic characteristic size, and finally, the matching graphs of the three polymers and the cores were formed. Through the three parallel core flooding experiments, the enhanced oil recovery effects of the different flooding systems were compared, and the reasons for the differences in the producing effects of the different layers were analyzed based on the parameters of the fractional flow rate and water cut. Finally, the optimal injection volume of the best EOR system was optimized and designed through the ultimate oil recovery and the EOR of the unit injection volume.

## 2. Material and Method

### 2.1. Materials

**Solution:** Three polymers and one surfactant were used to prepare the displacement solution. The polymers included a low-molecular-weight polymer (named LMP, with a molecular weight of 9 million Da), a high-molecular-weight polymer (named HMP, with a molecular weight of 25 million Da), and a hydrophobically associating polymer (named HAP, with a molecular weight of 6 million Da). The surfactant was a kind of petroleum sulfonate anionic surfactant. The polymer samples were non-toxic and tasteless dry powder (effective content 90%), the petroleum sulfonate (named PS) was a viscous flowable state (effective content 30%), and the above agents were provided by Daqing Refining and Chemical Company, Daqing, China. The polymers and surfactants needed to be prepared with a mother liquor with a mass concentration of 5000 mg/L and then diluted with simulated formation water to the target concentration. The four flooding systems were LMP solution, HMP solution, HAP solution, and LMP + PS binary solution.

**Crude oil and brine:** The inorganic salt was added proportionally to the deionized water (prepared by a UPT-I-10T Ultra-pure Water Purifier from Chengdu Youpu Super Pure Technology Co. (Chengdu, China)) to prepare the simulated formation water. The ion formula is shown in Table 1, with a total salty content of 5015.22 mg/L. The sodium chloride (NaCl), magnesium chloride (MgCl), sodium carbonate (NaCO_3_), et al. were purchased from Shanghai Aladdin Reagent Co. (Shanghai, China), and they were chemically pure. The degassed and dehydrated crude oil was provided by the Daqing Oilfield, China (9.68 cP at 50 °C).

**Models:** Cylindrical artificial cores with a diameter of 2.5 cm and a length of 30 cm and square artificial cores with a size of 4.5 cm × 4.5 cm × 30 cm were used in the injectivity experiments and oil displacement experiments, respectively. The effective permeabilities were about 50 mD, 170 mD, 210 mD, and 350 mD for the injectivity tests, and 50 mD, 170 mD, and 350 mD for the oil displacement experiments.

### 2.2. Properties of Polymers and Surfactant

#### 2.2.1. Viscosity Performance

The polymer mother liquors were diluted to 300 mg/L, 500 mg/L, 800 mg/L, 1000 mg/L, 1200 mg/L, 1500 mg/L, 1800 mg/L, and 2000 mg/L, respectively, and the viscosity was tested with a Brookfield viscometer (Middleboro, MA, USA) at a rotational speed of 6 rpm. The above target solutions (500 mg/L, 800 mg/L, 1000 mg/L, and 1500 mg/L) were sheared for 30 s with a Warring mixer at level 3 and the viscosity test was performed again. After shearing, it was necessary to wait for the bubbles generated in the solution to defoam before testing the viscosity.

#### 2.2.2. Rheological Properties

The rheological and viscoelastic curves of the polymer solutions with target concentrations of 500 mg/L, 800 mg/L, 1000 mg/L and 1500 mg/L were tested with a Haake rheometer (RS6000, produced in Thermo Fisher, Karlsruhe, Germany). The rheological curve adopted the shear mode, and the viscoelasticity test adopted the oscillation mode.

#### 2.2.3. Polymer Stability Evaluation

Formation water contains high-valent metal cations and has a high temperature, and the polymer solution will migrate and stain in the reservoir for a long time. Therefore, it was necessary to evaluate the salt resistance and aging stability of the polymer solutions. The evaluation was carried out based on the change in the viscosity of the polymer solution after aging. The polymer solution was diluted to 1000 mg/L with simulated water with a salinity of 0 mg/L, 7000 mg/L, 10,000 mg/L, 15,000 mg/L, and 20,000 mg/L, and the viscosity of the solution was tested with a Brookfield viscometer after standing for 2 days. The polymer solution was diluted with simulated formation water to a concentration of 500 mg/L, 1000 mg/L, and 1500 mg/L, respectively, and distributed in colorimetric bottles and aged in an oven at 45 °C. A colorimetric bottle was taken out at regular intervals and the viscosity of the solution was measured to calculate the viscosity retention rate. The aging time of the polymer required for this experiment was 5 days.

#### 2.2.4. Interface Tension Evaluation

The IFT of the HAP and LMP+PS binary system was tested by a rotating interfacial tensiometer (the DataPhysics surface interfacial tension meter is made in Karlsruhe, Germany) to evaluate the interfacial activity of the system. The concentration of LMP was 1000 mg/L, and the concentration of PS was 0 wt%, 0.05 wt%, 0.1 wt%, 0.2 wt%, and 0.3 wt%, respectively. The test temperature was 45 °C, and the test time was when the oil drop was broken or reached 2 h.

#### 2.2.5. Microporous Membrane Testing Hydrodynamic Characteristic Size

**Figure 1:**
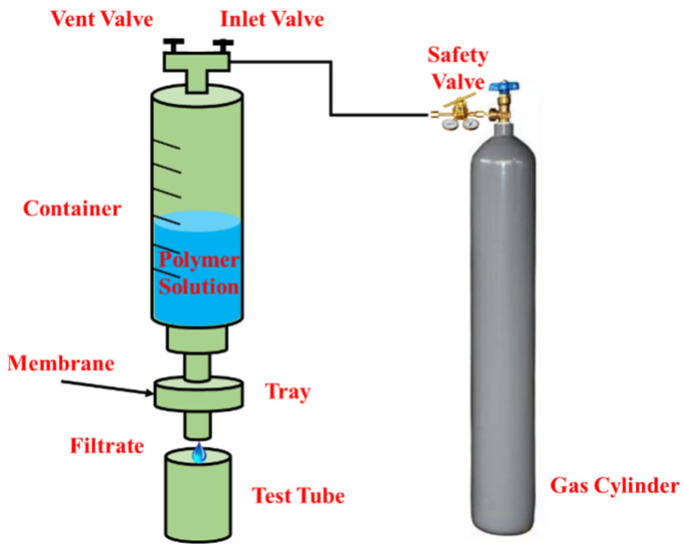
Flowchart of the polymer hydrodynamic characteristic size test.

According to the flowchart shown in Figure 1, the hydrodynamic characteristic size of the polymer solution was tested by the microporous membrane method. Aa gas cylinder was used to apply a pressure of 0.1 MPa, the filter container was filled with 200 mL of solution, the filter membrane (PVDF) was gradually reduced from 0.8 μm to 0.15 μm, and the filter membrane was replaced after each filter membrane flowed out 20 mL of solution. A Brookfield viscometer was used to measure the viscosity of the filtrate, and a relationship curve was drawn between the viscosity retention rate and the filter membrane size. Here, the filter membrane size corresponding to a viscosity retention rate of 80% was the polymer hydrodynamic size. The experiment was carried out at room temperature, and the concentration of each polymer was 500 mg/L, 800 mg/L, 1000 mg/L, and 1500 mg/L.

### 2.3. Injectivity of Polymers

**Figure 2:**
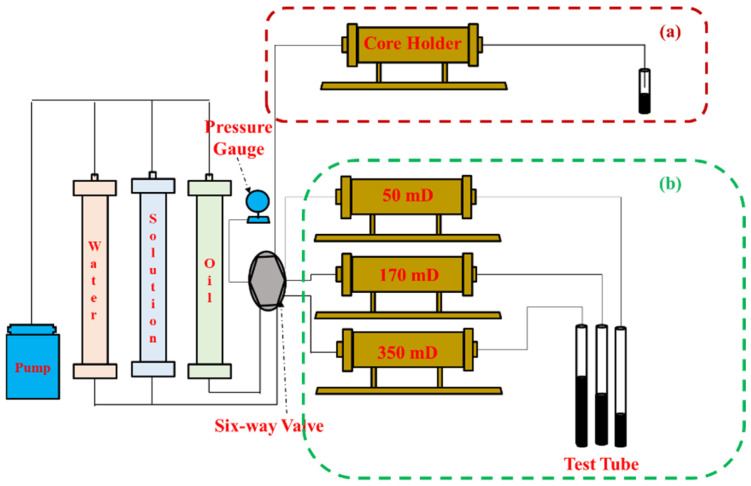
Flowchart of core flooding experiments: (**a**) injectivity experiments, and (**b**) oil displacement experiments.

The polymer injectivity was evaluated by the resistance factor (R_F_) and residual resistance factor (R_FF_). The experimental steps were as follows. ① Use the intermediate container to evacuate and saturate the core with water, and use the mass conservation method to calculate the pore volume. ② Connect the experimental device according to Figure 2a, use the simulated formation water to measure the permeability, and record ΔP_w_ after the pressure is stable. ③ Carry out polymer flooding, continue to inject 1–2 PV after the pressure is stable, and record the stable pressure ΔP_p_. ④ Carry out the second water flooding until the pressure is stable and record ΔP_w’_. The experiment was carried out in a 45 °C incubator, and the injection rate was 0.3 mL/min. The drag coefficient and residual drag coefficient were calculated according to Formulas (1) and (2).

(1) $$R_{F}=\frac{\lambda_{w}}{\lambda_{p}}=\frac{\Delta p_{p}}{\Delta p_{w}}$$

(2) $$R_{FF}=\frac{K_{w\prime }}{K_{w}}=\frac{\Delta p_{w\prime }}{\Delta p_{w}}$$

The polymer injectivity is the premise used to ensure full oil displacement performance. In Section 2.2.5, the hydrodynamic characteristic size test of the polymer was carried out, which can preliminarily judge the matching relationship between the polymer and the reservoir [31]. In order to reduce the work, the test sequence in the injection performance experiments in this section will start with the combination of a low polymer concentration and a high core permeability for the best injectivity. If a certain combination of concentration and permeability is mismatched, there is no need to conduct injectivity experiments with high-concentration or low-permeability cores. The specific experimental scheme is shown in Table 2.

### 2.4. Oil Displacement Design

#### 2.4.1. EOR Efficiency of Three Displacement Systems

The oil displacement experiment was carried out by using the three parallel core model to simulate the heterogeneity of the reservoir. The specific experimental steps were as follows. (1) Preparation stage: ① the core was vacuumed for 4 h, ② the core was self-absorbed and saturated with water for 2 h, ③ the water permeability was measured, and ④ the oil was saturated and aged for 2 days. (2) The displacement stage: the experimental flowchart was connected according to Figure 2b, and then the flooding process was water flooding until the comprehensive water cut reached 90%–chemical flooding to the set injection volume–subsequent water flooding until the comprehensive water cut reached 98%. The injection pressure and fluid production were recorded throughout the process. (3) Data processing stage: the variation curve of the displacement characteristic parameters (water cut, recovery factor, fractional flow rate) with the injection volume was drawn, and the influence of different variables on the enhanced recovery factor were compared and analyzed. From the experimental results in Section 2.3, it can be seen that the matching between the HMP polymer and the target permeability core is poor. Here, only the EOR effects of the LMP, HAP, and LMP-PS binary systems are compared. The specific experimental scheme is shown in Table 3 No. 1–3.

#### 2.4.2. Effect of Injection Volume on EOR

In Section 2.4.1, the best EOR displacement solution can be optimized. Theoretically, the larger the injection volume of the solution, the higher the ultimate recovery, but the cost of the chemical agent will also increase accordingly. Therefore, there is an optimal solution injection volume, which can obtain the best economic benefits. Here, the injection rate of the system is optimized through the three parallel core flooding experiments. The experimental procedure is the same as that in the Section 2.4.1. The specific experimental scheme is shown in Table 3, No. 4–6.

## 3. Results and Discussion

### 3.1. The Basic Properties of the Displacement Solution

#### 3.1.1. Viscosity Performance

**Figure 3:**
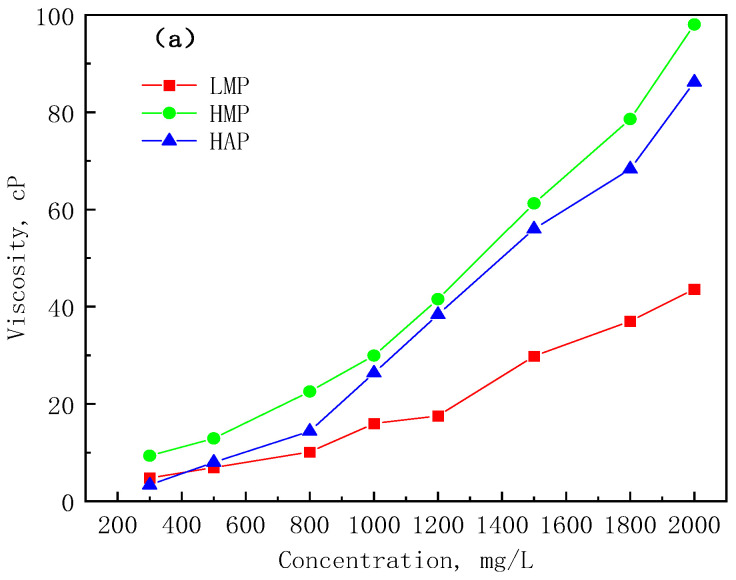

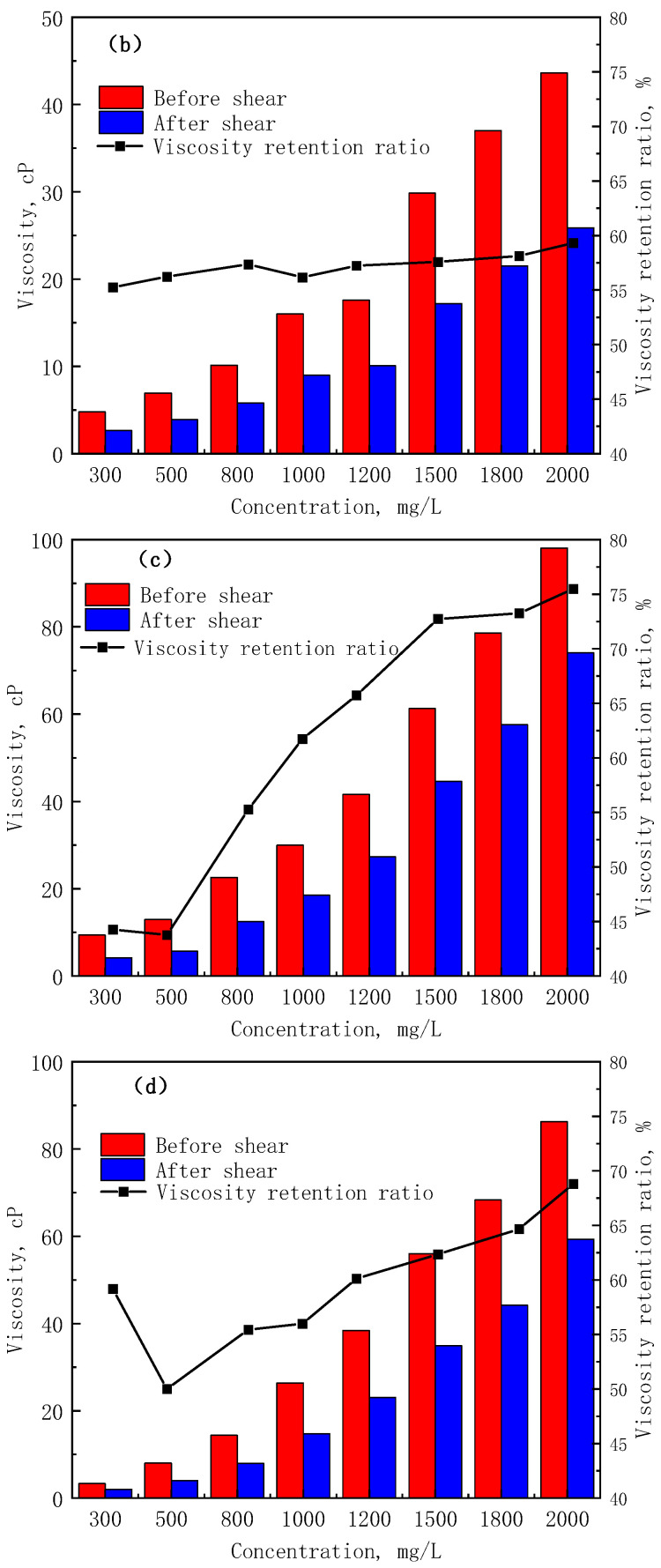
Viscosification properties and shear resistance properties of the polymer solutions. (**a**) Viscosity–concentration curve; (**b**) viscosity retention rate of LM; (**c**) viscosity retention rate of HM; and (**d**) viscosity retention rate of HAP.

The relationship curves of the polymer viscosities with the concentration of the solutions are shown in Figure 3a. The three polymers all have good viscosity-increasing properties, which will become stronger with the increase of the molecular weight, and a higher viscosity can be obtained under the premise of a lower molecular weight through hydrophobic association. The viscosity of the HMP and HAP solutions is much higher than that of the LMP solution at the same concentration higher than 1000 mg/L; the viscosity of the HAP polymer changes significantly when it is 800–1000 mg/L, and the critical association concentration can be preliminarily determined to be around 800 mg/L. Hydrophobic associations can dramatically increase solution viscosity, but at the same time, have a negative effect on injectability.

It can be seen from Figure 3 that the viscosity retention rates of the three polymers after shearing are all above 50%, and they have good shear resistance. The viscosity retention rate of the LMP changed little with the concentration; the retention rate of the HAP and HMP increased with the increase of concentration, and the increase rate of the HMP was larger. The above results show that the increase in the concentration and molecular weight will enhance the shear resistance of the solution, and the hydrophobic association can also effectively resist the shear effect.

#### 3.1.2. Rheological Properties

**Figure 4:**
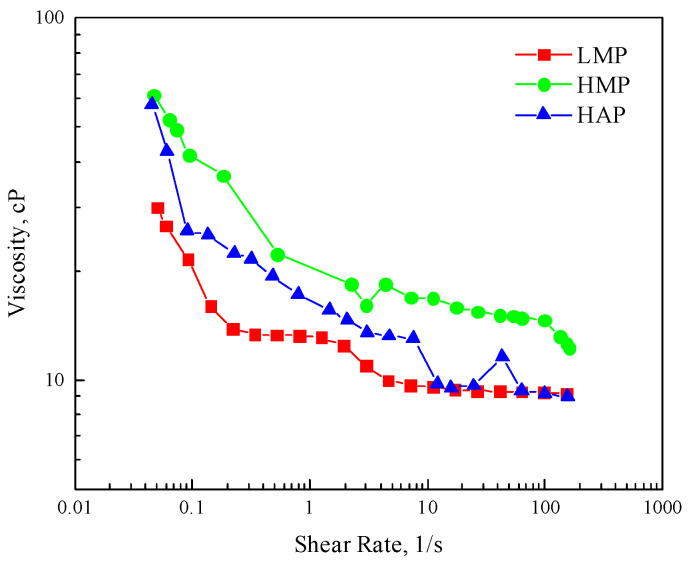
Comparison of the rheological curves of the three polymer solutions with a 1000 mg/L concentration.

The rheological curves (relationship curves of viscosity versus shear rate) of the polymer solutions with four concentrations are shown in Figure S1. The rheological curves of the polymer solutions are all of the power-law type, showing shear thinning characteristics. The viscosity of the polymer decreases rapidly when the shear rate is 1–4 1/s, and the apparent viscosity hardly changes with the increase of the shear rate after 10 1/s, that is, the second Newtonian zone appears. As the concentration increases, the viscosity of the polymer solution is less affected by the shear rate. Figure 4 shows the rheological curves of three polymer solutions with a concentration of 1000 mg/L. It can be seen that the rheological curves of the HAP polymers are between those of the LMP and the HMP, indicating that the increase in the molecular weight has a stronger impact on the shear resistance than the hydrophobic association.

**Figure 5:**
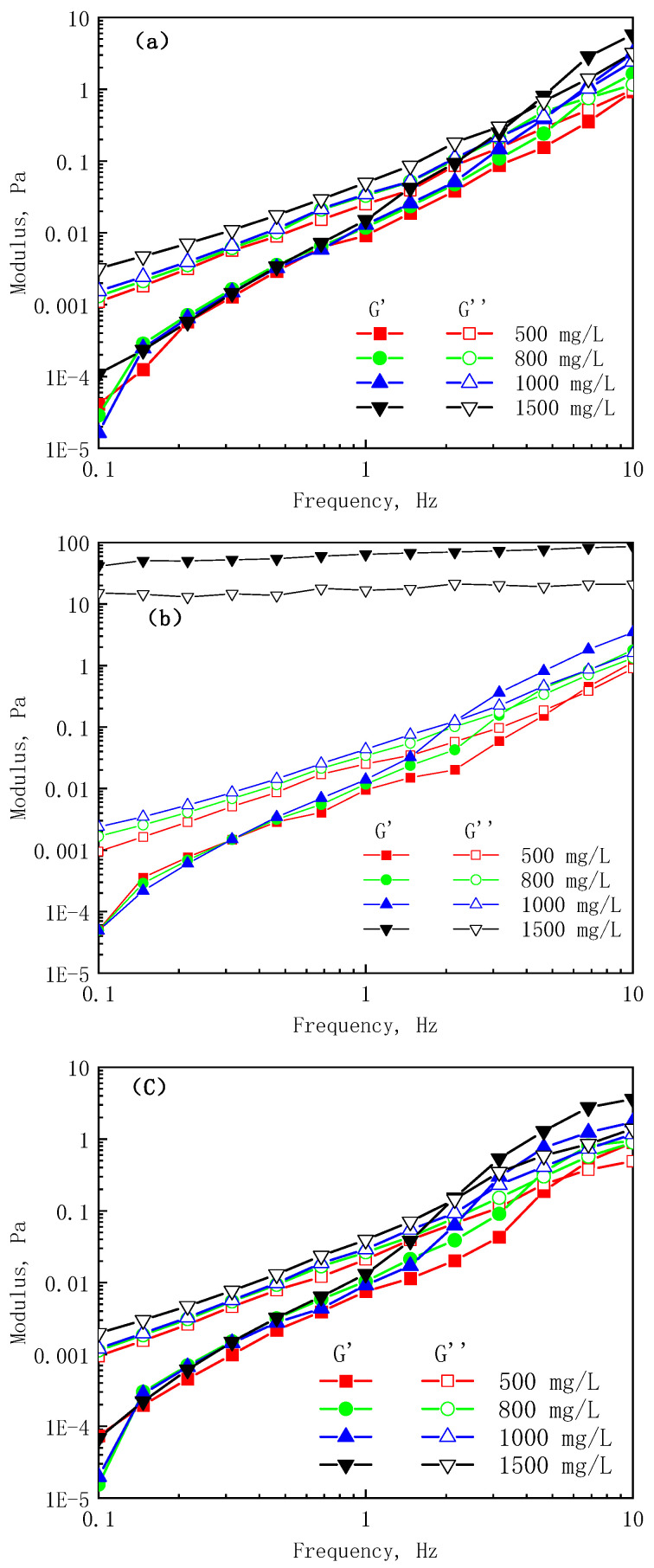
Viscoelasticity of the three polymers: (**a**) LMP, (**b**) HMP, and (**c**) HAP.

A polymer solution belongs to a viscoelastic fluid, which has both the elasticity of the solid and the viscosity of the liquid. Under the action of external force, polymer solutions can change between viscosity and elasticity. The elasticity and viscosity of viscoelastic fluids can be characterized by the storage modulus (G’) and loss modulus (G”), respectively. The change curves of the storage modulus (G’) and loss modulus (G”) in response to the angular frequency are shown in Figure 5.

Figure 5 shows that the change trends of the storage modulus and loss modulus of the polymer solution are consistent: with the increase of the angular frequency, the storage modulus and the loss modulus increase; at a low angular frequency, except for the HMP solution with a concentration of 1500 mg/L, the storage modulus is greater than the loss modulus, all of which are viscous; at high angular frequencies, the storage modulus is greater than the loss modulus, and the solution behaves elastically. The greater the mass concentration of the same polymer, the greater the storage modulus and loss modulus, and the viscoelasticity of the solution is enhanced.

**Figure 6:**
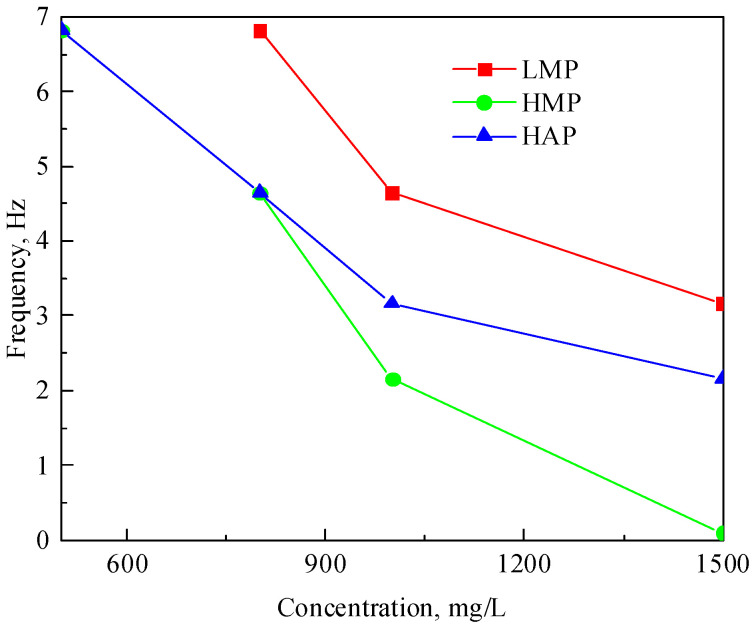
Relation curves of the viscoelastic critical frequency and concentration of the polymer solutions.

The storage modulus and loss modulus curves of the polymer solutions at different concentrations have an intersection point, and the relationship between the intersection point and the solution concentration of the three polymers is shown in Figure 6. When the angular frequency is lower than the angular frequency at the intersection point, the loss modulus is greater than the storage modulus, and the solution is mainly viscous; otherwise, the solution is mainly elastic. As the polymer molecular weight and solution concentration decrease, the angular frequency decreases accordingly. This is because the entanglement of the polymer molecules becomes severe with the increase of the molecular weight and concentration, and a stronger elastic effect can be exhibited at lower angular frequencies. The intersection angle frequency of the HAP is lower than that of the LMP because the hydrophobic association can greatly increase the viscoelasticity of the solution. However, when the concentration is greater than 800 mg/L, the intersection angle frequency of the HAP is higher than that of the HMP, indicating that at high concentrations, the molecular weight has a stronger influence on the solution viscoelasticity than the hydrophobic association.

#### 3.1.3. Polymer Stability

**Figure 7:**
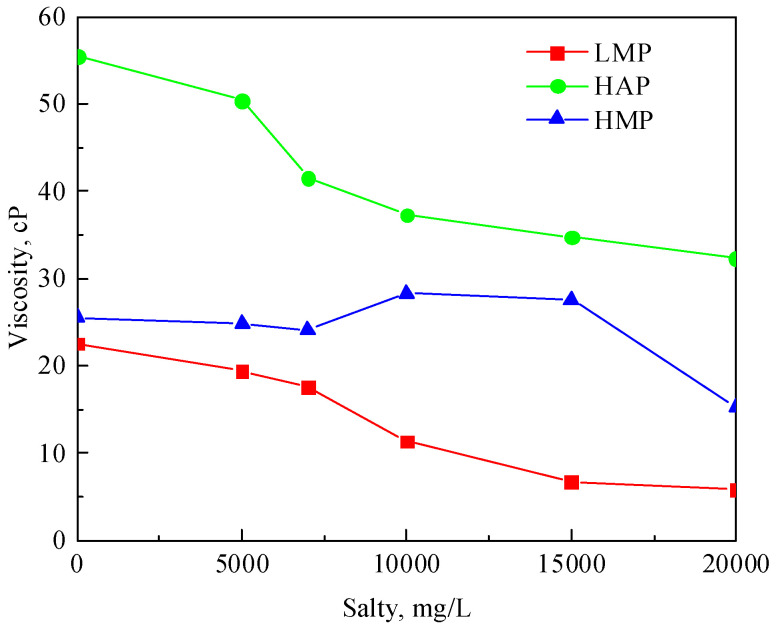
Variation curves of the viscosity with the salinity of the three polymers at 1000 mg/L.

The viscosity changes of the three polymer solutions with a concentration of 1000 mg/L in brine with different salinity are shown in Figure 7. Salinity has a great influence on the viscosity of polymer solutions. The viscosity of the LMP and HMP polymers gradually decreases with the increase in salinity. This is mainly because the addition of cations neutralizes the anions of the straight chain of the polymer, which reduces the electrostatic repulsion in the molecule, resulting in the curling of the polymer molecules and a poor viscosity-increasing effect. The structure of the HAP is more stable due to the introduction of hydrophobic groups on the polymer chain. In addition, due to the effect of the hydrophobic groups, the HAP molecules can form intermolecular and intramolecular associations. The water polarity will increase when the salinity increases, which can promote intermolecular associations. The viscosity of the HAP solution is affected by the intermolecular association and electrostatic action, so the viscosity first increases and then decreases.

**Figure 8:**
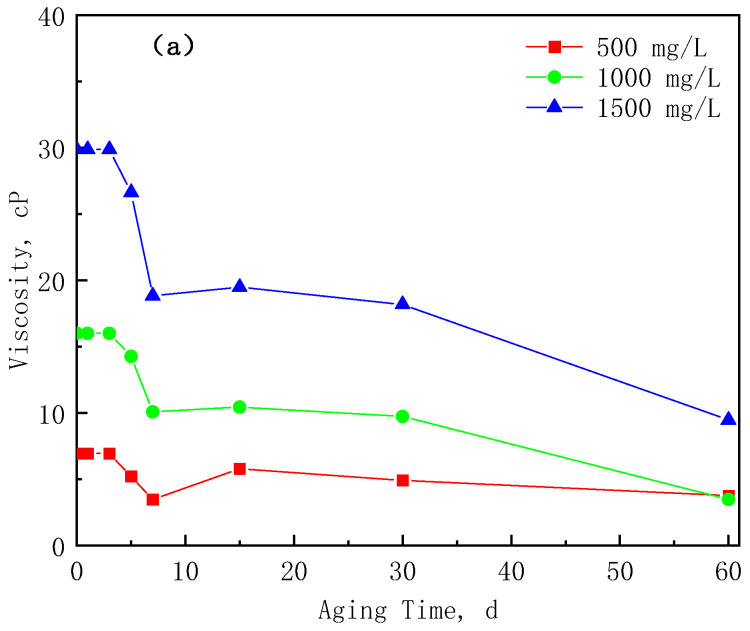

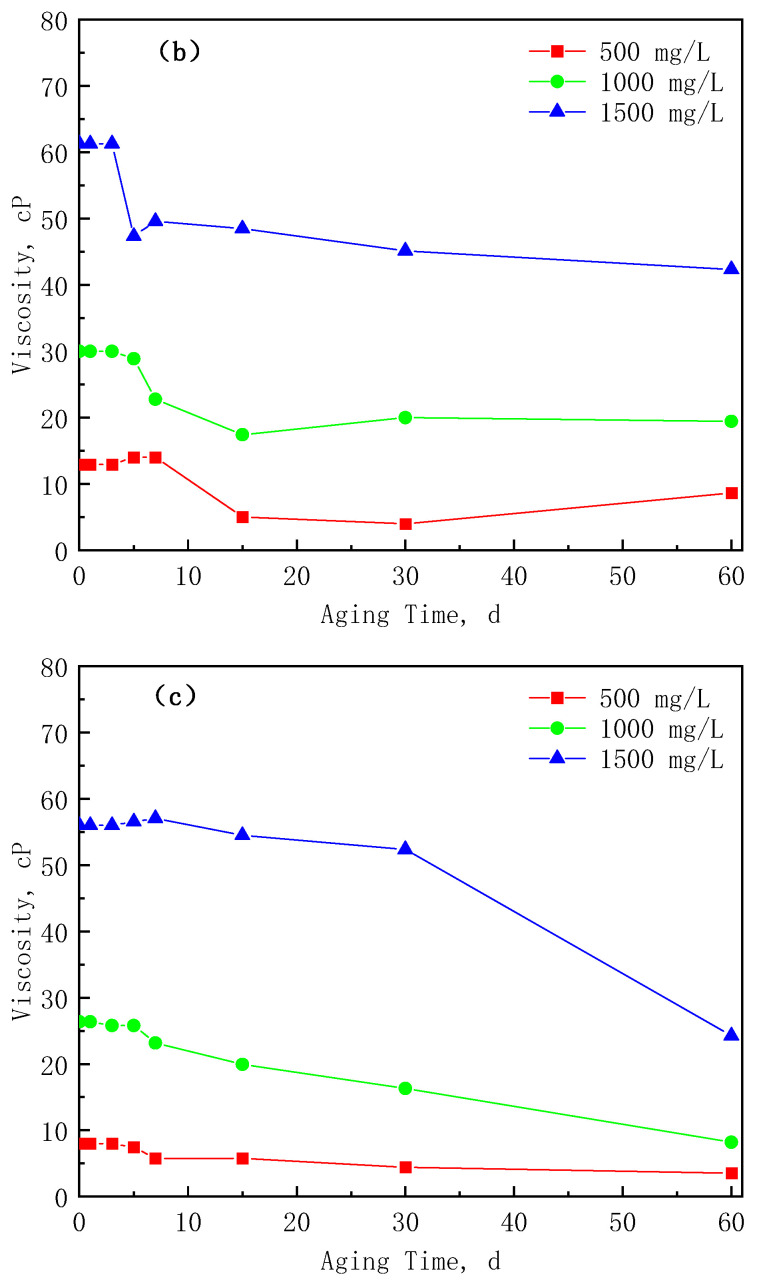
The viscosity of the three polymers as a function of the aging time: (**a**) LMP, (**b**) HMP, and (**c**) HAP.

The relationship between the viscosity retention rate and the aging time of the three polymers at three concentrations is plotted, as shown in Figure 8. Figure 8 shows that the viscosity of the LMP and HMP solutions hardly changes in the first three days, and the retention rate is above 90%; the viscosity drops rapidly on the 3rd to 7th day; the viscosity remains stable from 10 to 30 d, and the retention rate is above 50%. At 60 d, the viscosity retention rate of the HMP polymer is still above 60%, while the viscosity of the LMP polymer decreases significantly (the viscosity retention rate is only 21% at the concentration of 1500 mg/L). LMP mainly relies on its own linear chain structure to form a hydration layer to increase viscosity, while HMP can form a more stable spatial network structure through the intertwining of molecular chains, which becomes more stable with the increase of the concentration. The viscosity of the HAP polymer remains constant at a concentration of 500 mg/L, while at 1000 mg/L and 1500 mg/L, the solution’s viscosity rapidly decreases on the 7th day and the 30th day, respectively, indicating that the hydrophobic association polymer has a more strong aging stability.

#### 3.1.4. Interface Activity

The oil–water IFT results of the HAP, LMP, and four kinds of S/P systems are shown in Table 4. The HAP solution has a certain interface activity, which is related to the hydrophobic association functional group of the side chain and has good displacing and washing performance. The IFT of the S/P system can reach ultra-low in the order of 10–3 mN/m. It can also be judged from the time when the oil bead wire is pulled off that the increase in the PS concentration can enhance the ability of the S/P system to reduce the IFT. Although hydrophobically associated polymers have certain interfacial activity, their ability to reduce the IFT is much weaker than that of the S/P binary systems.

#### 3.1.5. Hydrodynamic Characteristic Size

The hydrodynamic characteristic size curves of the three polymers are shown in Figure S2, and the hydrodynamic characteristic sizes of each solution obtained according to the standard of 80% viscosity retention rate are shown in Table 5. The hydrodynamic characteristic size is affected by the solution concentration, molecular weight, and association. The hydrodynamic characteristic size is the equivalent particle size formed by the intertwining of polymer molecular coils. When the molecular weight increases, the concentration increases, and under the action of association, the chance and degree of interpenetration and overlapping of the polymer coils will increase, resulting in an increase in the molecular size.

**Figure 9:**
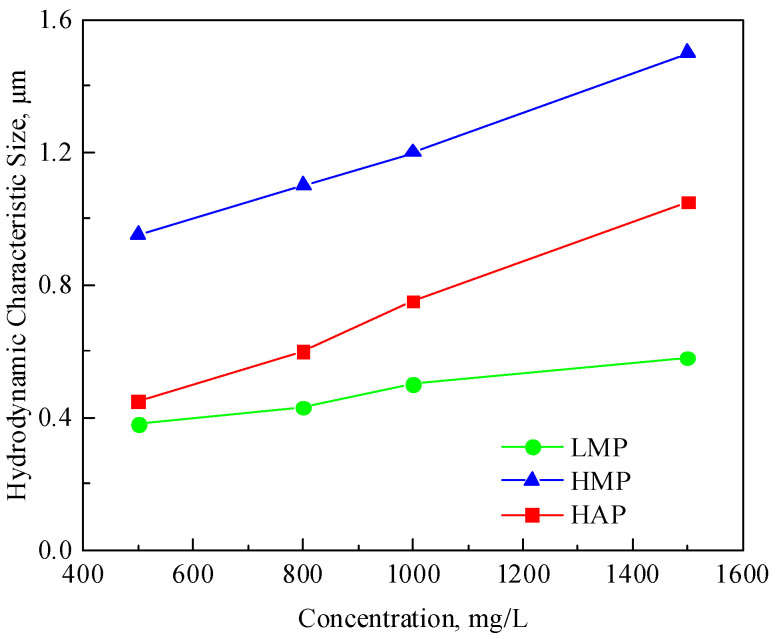
Variation curve of the polymer hydrodynamic size with the concentration.

The relationship curves of the hydrodynamic characteristic size and concentration of the three polymers are shown in Figure 9. The hydrodynamic characteristic size of HMP is much larger than that of LMP and HAP at the same concentration, indicating that the molecular weight has a greater influence on the characteristic size. This is because there are a large number of strongly polar -CONH_2_ and -COO-Na^+^ side groups on the long chain of the polymer molecule, the hydrogen bond is very strong, and physical cross-linking points are easily formed between the molecules, thus forming a spatial network structure. When the molecular weight of the polymer increases, the longer the molecular chain of the polymer is, the easier it is for the molecular chains in the aqueous solution to be entangled, and a more complex and stable network structure will be formed. Due to the introduction of branched functional groups, the HAP polymer can easily to form a spatial network structure and has a larger hydrodynamic characteristic size than LMP.

The larger the hydrodynamic characteristic size of the polymer, the stronger the flow resistance in the reservoir, which is beneficial for control of the profile. But, it will also increase its injection and flow difficulties in the reservoir, so the matching design of the polymer and the reservoir in Section 3.2 is required.

### 3.2. Matching Design of Polymer and Reservoir

The main mechanism for a polymer to enhance oil recovery is to improve the water–oil mobility ratio by increasing the viscosity of the water phase, but the excessive viscosity of the solution (or the large hydrodynamic characteristic size) will also lead to its weaker injectivity [2]. Therefore, for the target reservoir, the viscosity of the polymer solution has an optimal application range, which needs to be optimized.

#### Injectivity Performance Determines Upper Viscosity Limit

**Figure 10:**
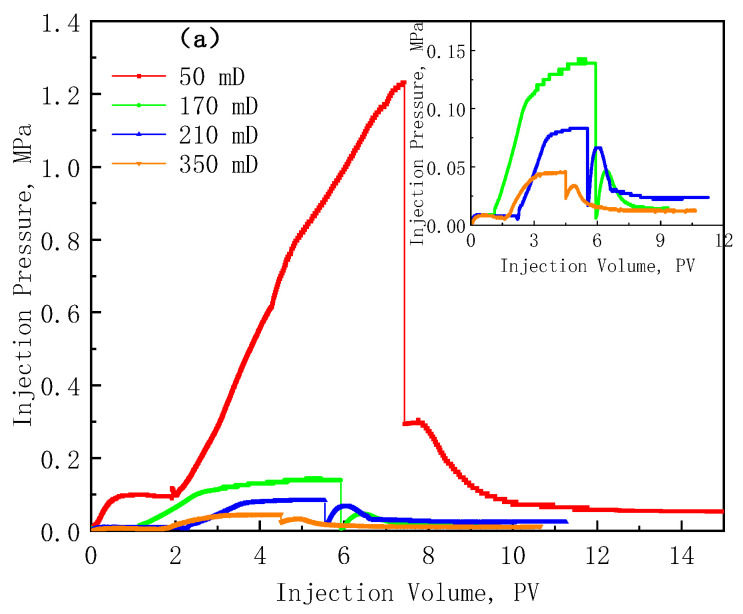

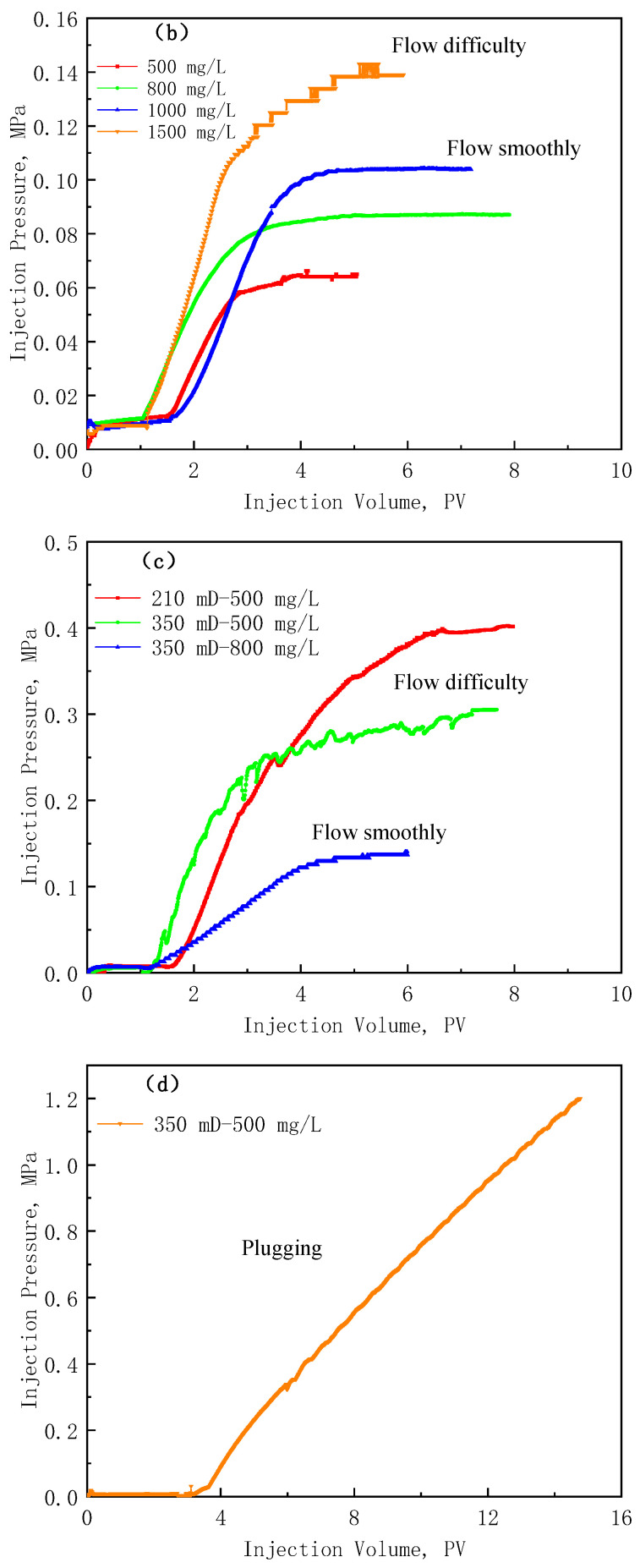
Pressure curve of the polymer injectivity experiments. (**a**) The injection pressure of 1500 mg/L LMP in four cores; (**b**) injection pressure of four concentrations of LMP solutions in 170 mD cores; (**c**) HAP injection pressure curve; and (**d**) HMP injection pressure curve. The injection pressure curves of the 21 groups of injectivity experiments listed in Table 2 are shown in Figure S3.

The upper limit of the application viscosity of the polymer solution is obtained by injectivity experiments. According to the experimental sequence of the optimal design in Section 2.3, the core flooding experiments of the three polymers with different concentrations were carried out sequentially, and the injection pressure curves are shown in Figure 10.

The injection pressure curves of the LMP polymer with a concentration of 1500 mg/L in the cores with the permeability of 50 mD, 170 mD, 210 mD, and 350 mD are shown in Figure 10a. It can be seen that when the permeability is 50 mD, the injection pressure rises rapidly within 5 PV of the injected polymer and there is no equilibrium trend, indicating that the injectivity of the solution in the core is poor at this time. When the permeability is greater than 170 mD, the injection pressure can be stabilized within 5 PV and kept at a low level, indicating its good injectivity. Figure 10b shows the injection pressure curves of four concentrations of LMP solutions in the 170 mD cores. It can be seen that once the high concentration of polymer has a good match with the core, reducing the polymer concentration also has a good matching relationship. The injectivity of the HAP polymer is poor, and only the injection pressure of the 500 mg/L solutions in the 210 mD and 350 mD cores and the 800 mg/L solution in the 350 mD cores can reach equilibrium, as shown in Figure 10c. Figure 10d is the injection pressure curve of the lowest concentration (500 mg/L) HMP polymer in the highest permeability (350 mD) core, and there is still no equilibrium trend after injecting 10 PV, indicating that the reservoir matching of HMP is the worst.

LMP has a low molecular weight and the lowest viscosity at the same concentration, so it has the best reservoir-matching relationship. HAP and MHP have similar viscosities at the same concentration, but a large difference in injectivity, which can be explained by the hydrodynamic characteristic size. MHP forms a spatial network structure through longer molecular chains being intertwined and stronger hydrogen bonds, and its hydrodynamic characteristic size is the largest; HAP forms a spatial network structure through hydrophobic association, and its hydrodynamic characteristic size is significantly larger than that of LMP, but still smaller than MHP, so its injection performance is in between.

As the polymer concentration increases and the core permeability decreases, the time required for the injection pressure curve to reach equilibrium increases gradually, which also means that the matching relationship between the two gradually becomes worse. The key parameters of the 16 groups of LMP injection experiments are shown in Table 6. It can be seen that the resistance factors of the 16 experiments are all within 20. This is because the core permeability is low and the base pressure difference of water flooding is high, resulting in the overall low resistance factor calculated in the end. So, the matching relationship between the polymer solutions and the cores cannot be judged by the value of the resistance factor.

**Figure 11:**
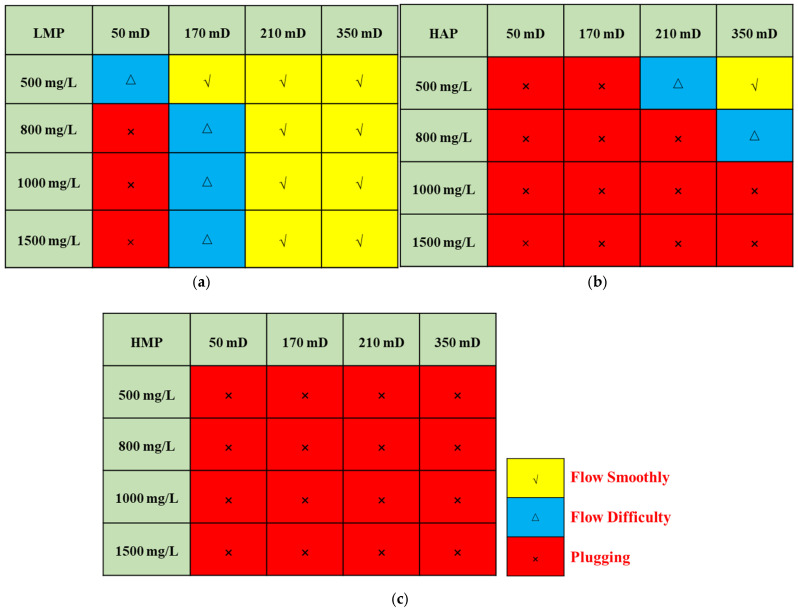
Three polymer reservoir-matching graphs: (**a**) LMP; (**b**) HAP; and (**c**) HMP.

Here, the matching relationship between the polymer and the core is divided by the injection volume required for the polymer injection pressure to reach equilibrium: the injection pressure has no equilibrium trend within 5 PV, representing that the matching relationship is plugging; the injection pressure reaches equilibrium within 3–5 PV, representing that the matching relationship is flow difficulty; the injection pressure is balanced within 3 PV, representing that the matching relationship is flow smoothly. Therefore, the reservoir-matching graphs of the three polymers can be obtained, as shown in Figure 11. According to the polymer–reservoir matching graphs, the upper limit of the concentration can be determined according to the permeability of the reservoir, which can avoid the near-well plugging of the polymer and reduce the cost.

### 3.3. Contrast in EOR Efficiency of Three Oil Displacement System

**Figure 12:**
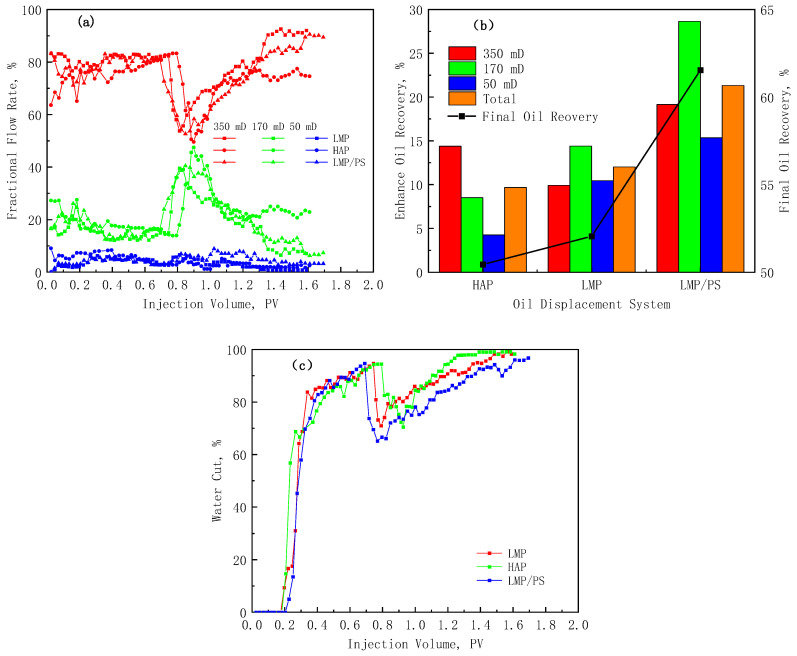
Displacement characteristic parameter curves of the three displacement solutions. (**a**) Fractional flow rate curve; (**b**) EOR and ultimate recovery of each layer; and (**c**) water cut curve.

The displacement characteristic parameter curves of the three displacement solutions are shown in Figure 12. Figure 12a shows the fractional flow rate curves of the cores in each layer. It can be seen that the fractional flow rate of the high-permeability layer is significantly reduced after the polymer and S/P systems are injected. Due to the high viscosity and large hydrodynamic characteristic size of the HAP polymer, the reduction of fractional flow rate in the high-permeability layer is more obvious and remains for longer. The fractional flow rate of the high-permeability layer during the S/P slug is significantly lower than that during the LMP slug. This is mainly because the S/P system has low IFT, which can generate emulsion in the core (Figure S4) and increase the flow resistance of the high-permeability layer. The maximum profile improvement rates obtained by the three solutions can be calculated by Equation (3) as 75.04%, 80.27%, and 74.57%, respectively. The profile improvement effect of HAP is the strongest, while LMP mainly plays the role of profile control during LMP flooding and S/P flooding. Emulsification often occurs in the middle and late stages of S/P flooding, so the maximum profile improvement rates of the two are similar, but the S/P system has a longer profile adjustment period.

(3) $$\lambda =\frac{Q_{hb}/\left(Q_{\mathrm{mb}}+Q_{\mathrm{lb}}\right)-Q_{ha}/\left(Q_{\mathrm{ma}}+Q_{\mathrm{la}}\right)}{Q_{hb}/\left(Q_{\mathrm{mb}}+Q_{\mathrm{lb}}\right)}$$

Figure 12b shows that although the HAP polymer has the best profile improvement effect, its final recovery and EOR efficiency are the lowest, which are 50.44% and 9.68%, respectively. This phenomenon can be explained by the heterogeneity of hydrophobically associated polymers [40]. There are differences in the degree of polymerization between the HAP molecules, and the solution is in a non-uniform state. Due to the large hydrodynamic characteristic size and viscosity of HAP, it promotes the solution to enter the middle- and low-permeability layers while generating high flow resistance in the high-permeability layer. This is the reason for the improvement of the fractional flow rate. At this time, the small-sized molecular coils generated by the heterogeneous association are more likely to enter the middle- and low-permeability layers, and the effect of enhancing oil recovery is poor. The LMP solution is a homogeneous system, which can enter the middle- and low-permeability layers under a relatively high-pressure gradient, and it has a better oil displacement effect. Figure 12c shows that the LMP/PS flooding can obtain the lowest water cut and the longest duration, which is reflected in the highest final oil recovery and enhanced recovery, which are 61.54% and 21.32%, respectively. The oil recovery enhancing layer of the LMP and SP is mainly the medium-permeability layer, while the main effect layer of the HAP in improving oil recovery is the high-permeability layer.

To sum up, although HAP has a high viscosity and certain interfacial activity, its compatibility with reservoirs is poor, and it cannot exert its good oil displacement advantages. But it still has a good application prospect in high-permeability reservoirs (1000–4000 mD) [40].

### 3.4. Optimization of S/P Injection Volume

**Figure 13:**
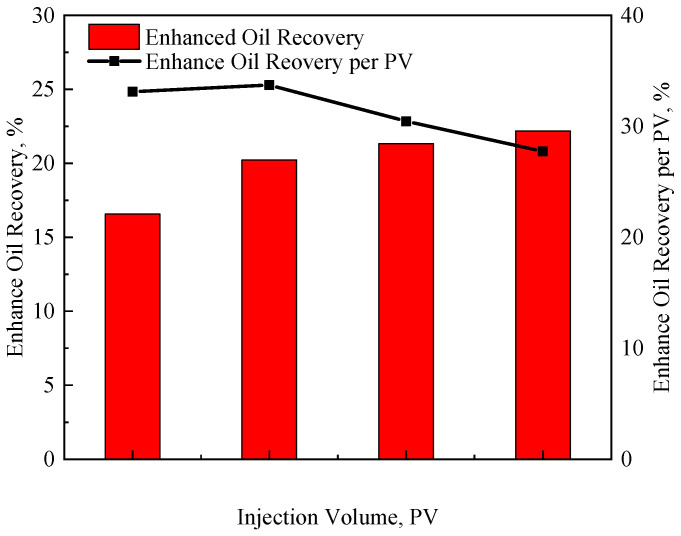
Comparison of the ultimate oil recovery and EOR of the unit PV in the four injection volume experiments.

The oil recovery curves of S/P flooding with four injection volumes are shown in Figure S5. As the injection rate increases, the ultimate recovery factor and EOR continues to increase, but the growth rate slows down. The optimal injection volume should be determined by comprehensively considering the EOR efficiency of the S/P solution and the ultimate oil recovery. Here, the enhanced oil recovery range of the unit PV flooding system is defined as the evaluation index to optimize the injection volume. Figure 13 shows the ultimate recovery and the unit PV agent EOR of the four injection volume experiments. It can be seen that when the injection volume increases from 0.5 PV to 0.6 PV, the ultimate recovery increases from 16.56% to 20.23%, an increase of 22.16%; and when the injection volume continues to increase from 0.7 PV to 0.8 PV, the ultimate recovery increases are, respectively, 5.34% and 4.08%, and the growth rates are significantly lower. The EOR value of the unit PV agent also reaches the maximum when the injection volume is 0.6 PV, which is 33.68%, and then decreases with the increase of the injection volume. Comprehensively considering the EOR efficiency (that is, economy) of the chemical agents and the ultimate oil recovery, the optimal injection volume of the S/P system should be around 0.6–0.7 PV.

## 4. Conclusions

In this paper, by comparing the viscosity-increasing performance, hydrodynamic characteristic size, injection performance, and enhanced oil recovery effect of LMP, HMP, and HAP, the effects of the molecular weight and association on the matching relationship between the polymer and the reservoir were studied, and finally, the injection parameters of the solution were optimally designed. The specific conclusions are as follows.

1. HMP and HAP have similar and better viscosity-increasing performance, shear resistance, and viscosity stability than LMP. When the concentration is higher, HMP exhibits stronger elastic properties than HAP, and the hydrodynamic characteristic size of HMP is obviously larger than that of HAP and LMP.
2. Both a large molecular weight and association will increase the flow resistance by increasing the solution viscosity and molecular coil size, but meanwhile, they will reduce the injection performance. According to whether the injection pressure curve reaches equilibrium and the time required for equilibrium, the degree of matching between the polymer solution and the reservoir can be divided into plugging, flow difficulty, and flow smoothly. The upper limit of the polymer concentration used in the reservoir of the target block can be obtained according to the matching graphs of the polymer and the reservoir: the upper limit of the concentration of LMP can reach 1500 mg/L, the concentration of HAP needs to be less than 800 mg/L, and the concentration of HMP needs to be lower. It provides a theoretical basis and corresponding reference data for the applicability of polymer systems under different physical property conditions.
3. Based on the mobility control theory, a method for designing the minimum polymer concentration was established. The water saturation range of the minimum mobility of the target core is between 30% and 40%. Therefore, for the cores of 210 mD and 350 mD, the concentration of the LMP polymer should be above 1000 mg/L and 1500 mg/L; the concentration of MAP should be 800 mg/L.
4. The HAP polymer has the best profile improvement effect, which mainly improves the recovery of the high-permeability layers, and the lowest EOR is 9.68%; the LMP profile improvement effect is worse than that of HAP, but it can produce more remaining oil in the middle-permeability layer, and the EOR can reach 12.01%; the EOR of the LMP/PS system is the highest at 21.32%, which can give full play to the oil displacement performance of the polymer and the oil-washing ability of the surfactant, and more middle-, high- and low-permeability layers can be produced. Meanwhile, the emulsification effect also makes the profile improvement effect last longer. Therefore, it provides a corresponding guiding basis for the use of polymer displacement under reservoir conditions with strong heterogeneity.
5. The best injection volume of LMP/PS can be optimally designed to be 0.6–0.7 PV according to the EOR efficiency (more than 20% at this time) and ultimate recovery of the unit PV agent (more than 30% at this time). Therefore, it provides certain data guidance for the efficient utilization of LMP/PS.

## Figures and Tables

**Table 1:** The formula for the formation water.

Ions	CO_3_^2−^	HCO_3−_	Cl	1/2 SO_4_^2−^	Ca^2+^	Mg^2+^	K^+^ + Na^+^	Total	pH
Concentration/mg/L	255.09	2334.02	815.35	14.41	34.07	10.94	1551.35	5015.22	8.49

**Table 2:** Experimental scheme of the polymer injectivity.

Number	Polymer	Concentration, mg/L	Permeability, mD	Note
1–14	LMP	500, 800, 1000, 1500	50, 170, 210, 350	Total in 14 experiments
15	HMP	500	350	Total in 1 experiment
16–21	HAP	500, 800, 100	170, 210, 350	Total in 6 experiments

**Table 3:** Parameters of the oil displacements.

Number	Cores Permeability	Polymer or S/P	Concentration, mg/L	Injection Volume, PV	Factor
1	About 50 mD–170 mD—350 mD, the error is within 10%.	LMP	1500	0.7	Displacement system
2		HAP	800	0.7	
3		LMP-S/P	1500–2000	0.7	
4		LMP-S/P	1500–2000	0.5	Injection volume
5		LMP-S/P	1500–2000	0.6	
6		LMP-S/P	1500–2000	0.8	

**Table 4:** Interfacial tension of the S/P binary system.

Polymer and Concentration	Surfactant	Concentration, %	IFT, mN/m	Notes
HAP, 500 mg/L	/	/	10.52	Not pull off
HAP, 1000 mg/L	/	/	5.39	Not pull off
HAP, 1500 mg/L	/	/	3.37	Not pull off
LMP, 1000 mg/L	PS	0	17.12	Not pull off
		0.05	0.0088	Not pull off
		0.1	0.0015	2 h pull off
		0.2	0.00454	1 h pull off
		0.3	0.00292	1 h pull off

**Table 5:** Hydrodynamic characteristic sizes of three polymers.

Polymers	Concentration, mg/L	Hydrodynamic Characteristic Size, μm
LMP	500	0.38
	800	0.43
	1000	0.50
	1500	0.73
MAP	500	0.45
	800	0.6
	1000	0.75
	1500	1.05
HMP	500	0.75
	800	1.10
	1000	1.20
	1500	>1.20

**Table 6:** Experimental results of the LMP injectivity experiments.

Concentration	50 mD		170 mD		210 mD		350 mD	
Permeability	R_F_	Balance Time, PV	R_F_	Balance Time, PV	R_F_	Balance Time, PV	R_F_	Balance Time, PV
500 mg/L	7.3	5	6.2	1.5	5.6	1.5	4.5	1
800 mg/L	/	Unbalanced	8.2	2	8.1	2	6.8	2
1000 mg/L	/	Unbalanced	10.3	2.5	10	2	8.5	2
1500 mg/L	/	Unbalanced	15.4	3.5	14.7	3	10.5	2.5

## Data Availability

The original contributions presented in this study are included in the article and Supplementary Materials. Further inquiries can be directed to the corresponding authors.

## Supplementary Materials

The following supporting information can be downloaded at: https://www.mdpi.com/article/10.3390/polym17101390/s1, Figure S1. Polymer Rheological Properties. (a) LMP, (b) HMP, (c) HAP; Figure S2. Curve of polymer viscosity retention versus membrane size. (a) LMP, (b) HMP, (c) HAP; Figure S3. The injection pressure of polymer injectivity experiments. (a) LMP-50 mD, (b) LMP-170 mD, (c) LMP-210 mD, (d) LMP-350 mD, (e) HAP, and (f) HMP; Figure S4. Emulsification of produced fluid during S/P flooding; Figure S5. Oil recovery curves of four injection volumes.
