# Supplementary material for: Reservoir Compatibility and Enhanced Oil Recovery of Polymer and Polymer/Surfactant System: Effects of Molecular Weight and Hydrophobic Association
Source: Polymers (Basel). 2025 May 18;17(10):1390. doi: 10.3390/polym17101390 (PMC12114843; doi:10.3390/polym17101390)
Supplement: Supplementary file 1 [file polymers-17-01390-s001.zip › polymers-3622037-supplementary.pdf]

Supplementary Material

Reservoir Compatibility and Enhanced Oil Recovery of Polymer and Polymer/Surface System: Effects of Molecular Weight and Hydrophobic Association

[a] College of Earth Sciences and Engineering, Xi'an Shiyou University, Xi'an 710065. China

[b] Shaanxi Key Laboratory of Petroleum Accumulation Geology, Xi'an Shiyou University, Xi'an 710065, China

[c] College of Petroleum Engineering, Xi'an Shiyou University, Xi'an 710065. China

[d] China National Oil and Gas Exploration and Development Company Ltd., Beijing 100034, China

[*] Corresponding author: *Tao Liu*, lt2019310174@163.com; t49806705@gmail.com

Tao Liu[a,b *], *Xin Chen*[c *], *Xiang Tang*[d]

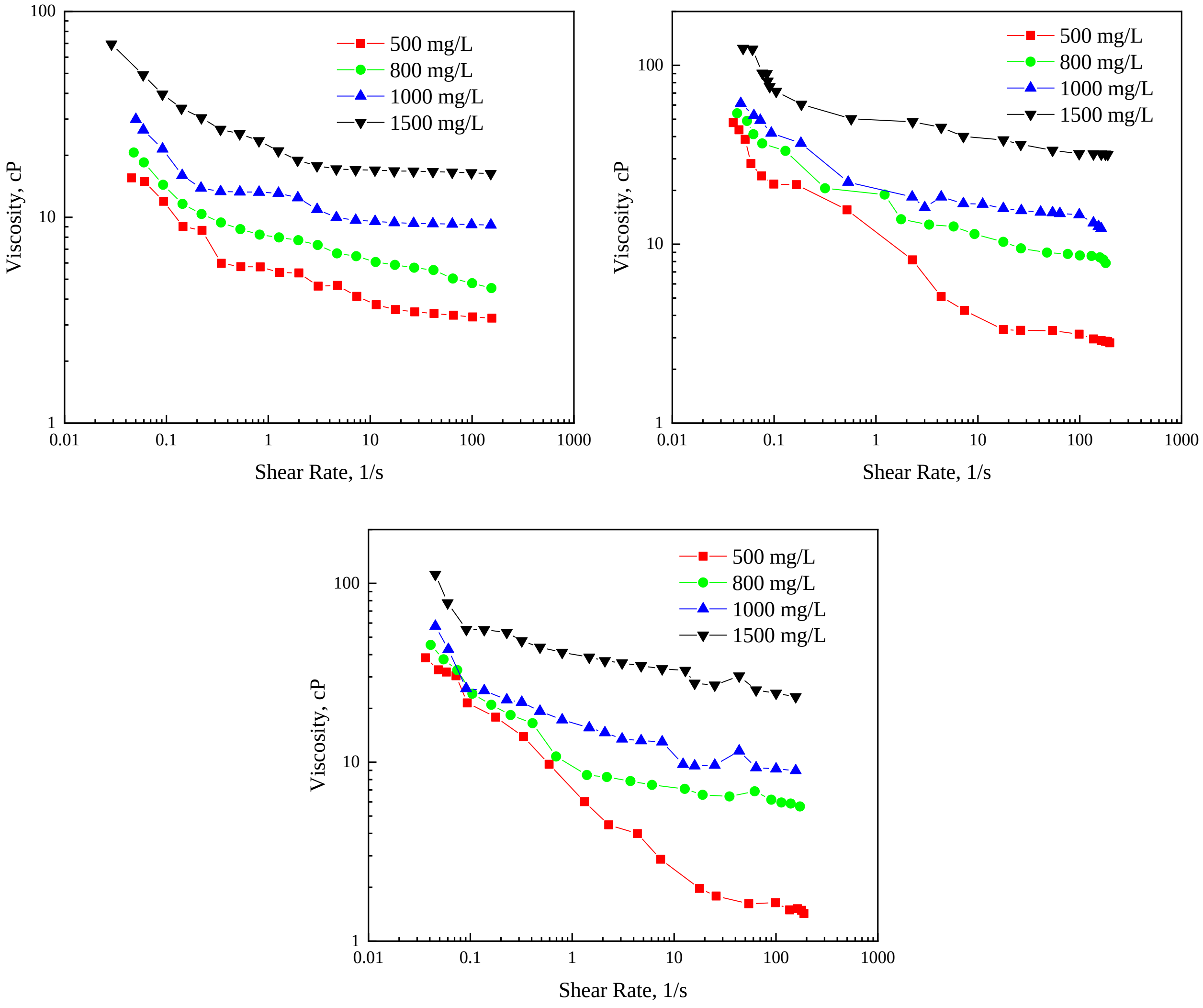

Figure S1. Polymer Rheological Properties. (a) LMP, (b) HMP, (c) HAP

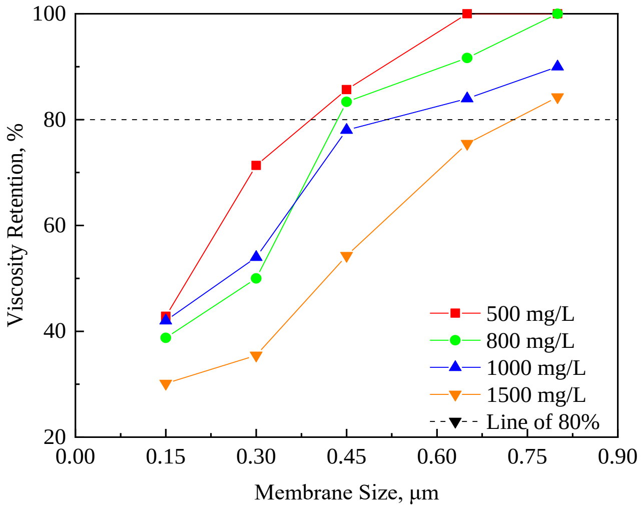

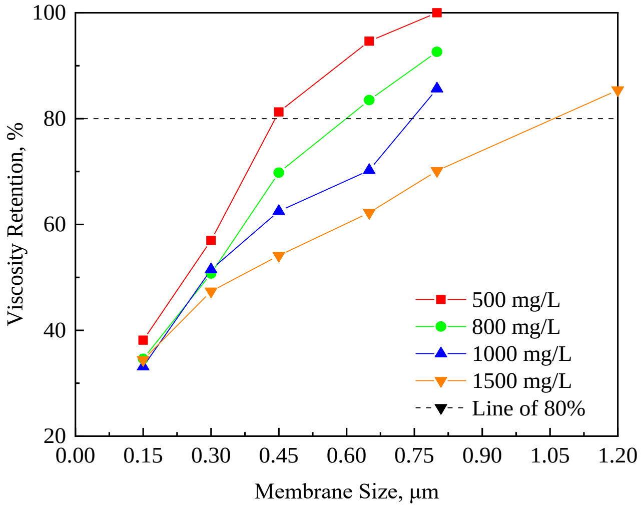

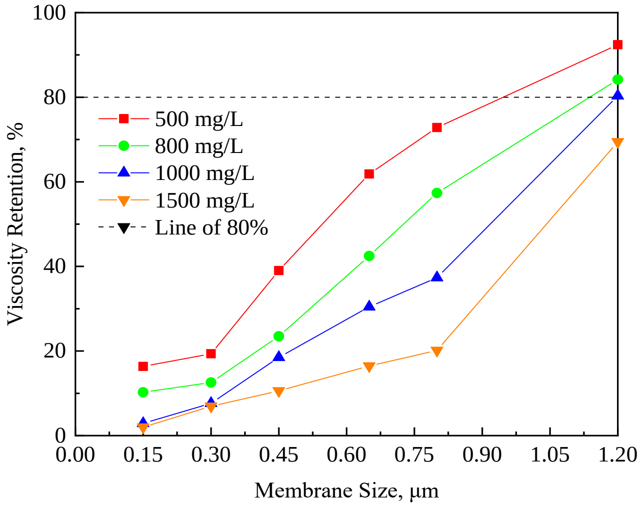

Figure S2. Curve of polymer viscosity retention versus membrane size. (a) LMP, (b) HMP, (c) HAP.

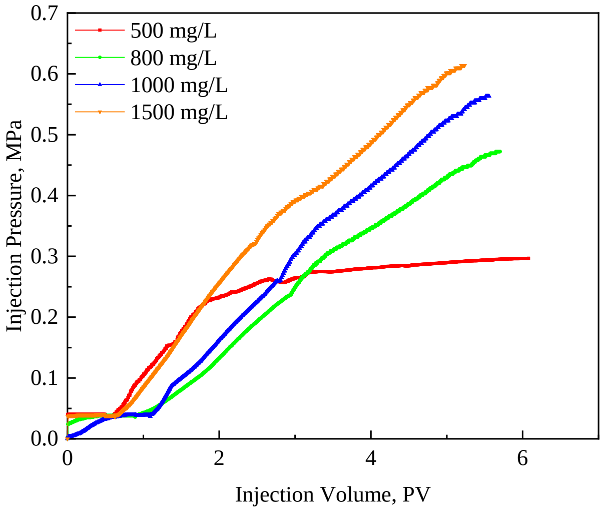

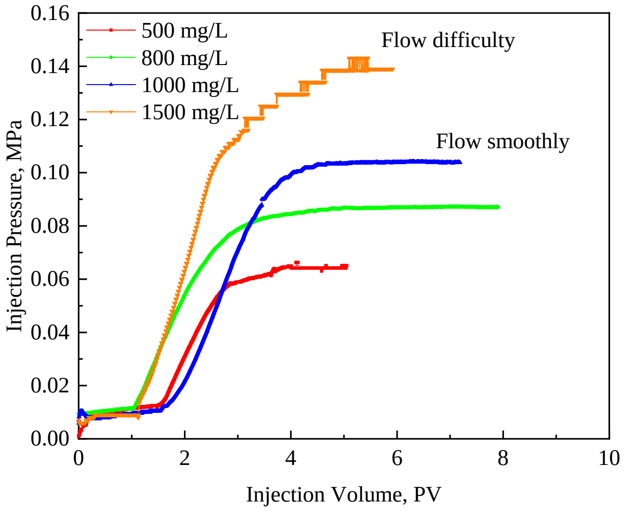

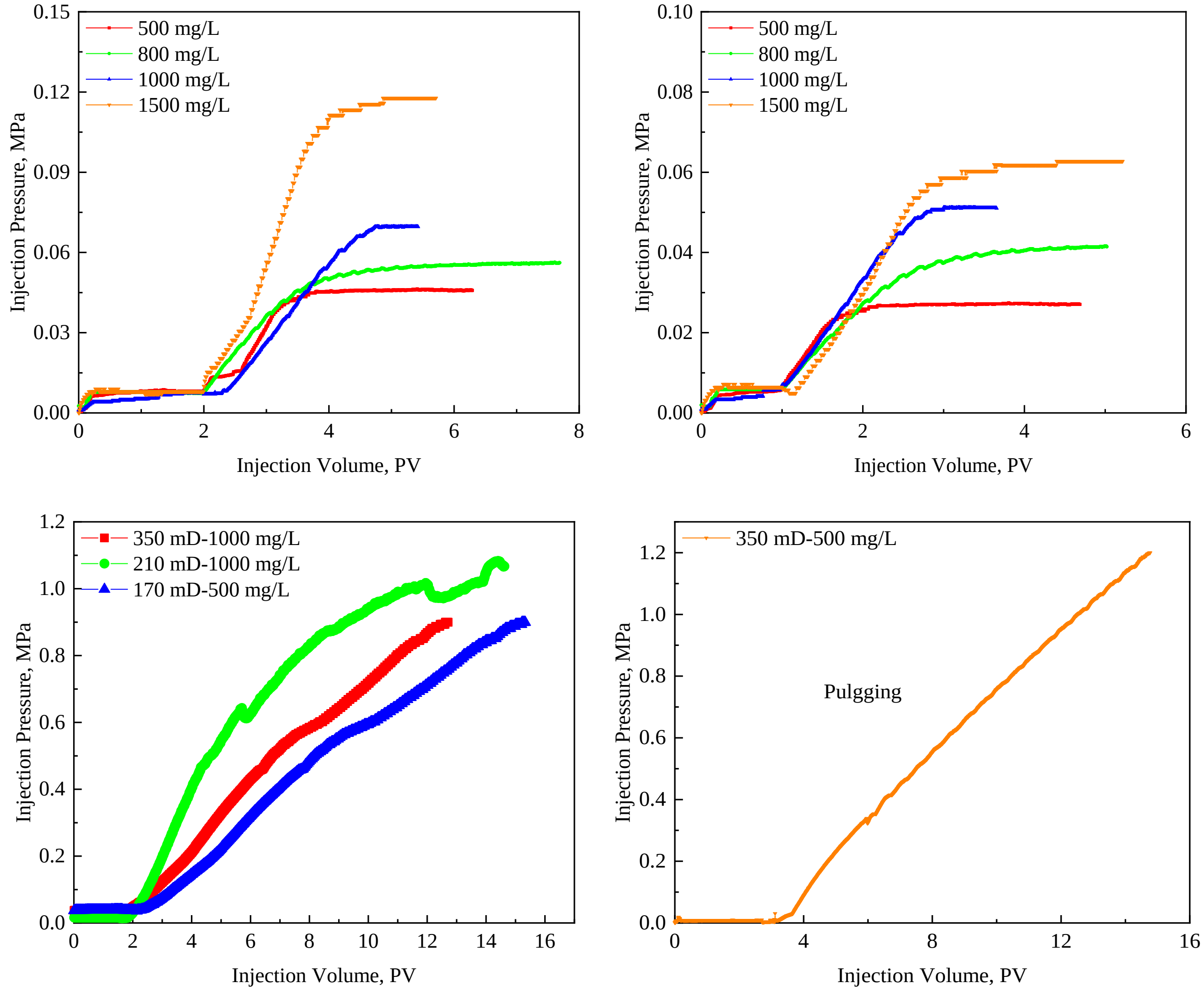

Figure S3. The injection pressure of polymer injectivity experiments. (a) LMP-50 mD, (b) LMP-170 mD, (c) LMP-210 mD, (d) LMP-350 mD, (e) HAP, and (f) HMP.

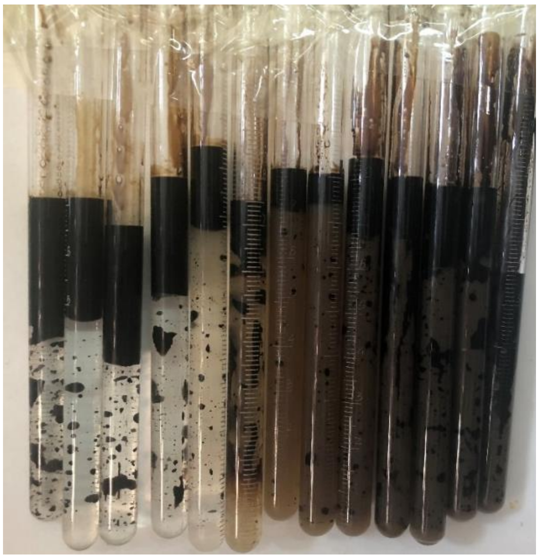

Figure S4. Emulsification of produced fluid during S/P flooding

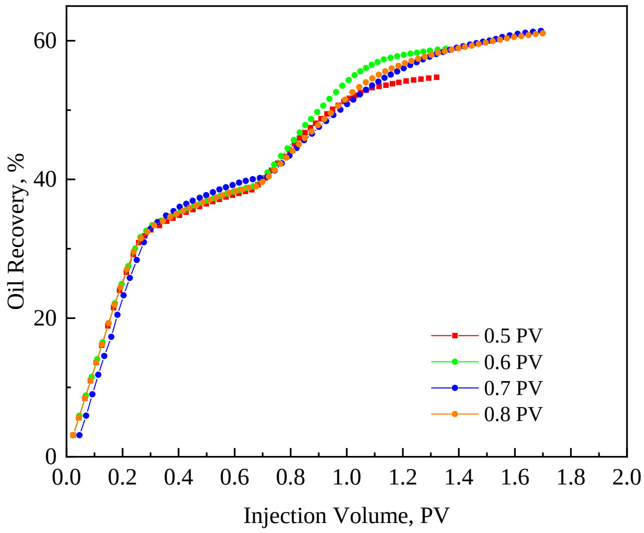

Figure S5. Oil recovery curves of four injection volumes
